# Around the Hospitals

**Published:** 1893-08-26

**Authors:** 


### Around the Hospitals.

Notice to the Managers of Institutions.—Special space is now reserved for the insertion of notices of hospital meetings and festivals. The Editor requests that all notices may reach The Hospital Office, 428, Strand, at least one week before the date of each meeting.

**The Royal Hants County Hospital.**—Not many of the indigent army of hospitals are in the satisfactory financial condition as the Royal Hants Hospital at Winchester, which, with a good balance in the banker's and matron's hands, has yet a large sum to invest for the building up of dependable income or the needs of rainy days. The number of in-patients treated during last year was 574, and out-patients 658. These numbers are far more evenly balanced than is usual even in country districts. At the instigation of the medical staff improvements have been made in the sanitary arrangements of the special wards. It has been arranged that the medical staff should be invited to attend the weekly meetings of the board of management. We notice that her Majesty the Queen has shown her approval of the hospital's work by a yearly subscription of £100, and a donation of £300 to the building fund.

**The London Homœopathic Hospital.**—This institution, which in its old quarters is already a thing of the past, is nevertheless carrying on a large amount of work in its temporary quarters in Powis Place, formerly the nursing institute of the hospital. Whilst receiving every acknowledgment that its services are both required and appreciated by the large sums subscribed towards the building fund, the subscriptions to the hospital for general purposes are not sufficient to meet its wants. Consequently, a reduction in the number of patients received was made last year. The authorities of the Homœopathic Hospital have an opinion that a large debt is not so desirable as many other hospitals think it. Here is an opportunity for those who appreciate such prudence to reward it by removing the debt on the hospital accounts altogether. They amount to upwards of £600. Mr. Cross, the Secretary-Superintendent, makes out a very good case for the hospital through the action of the Hospital Sunday and Saturday Funds. Both, acting on different methods of distribution, are equally complimentary to the management. It will be remembered that the foundation stone of the new building was recently laid by the Duchess of Teck. It is to afford accommodation for one hundred patients, and promises to be a complete and handsome building. It is expected that about £10,000 more will be required to carry out the designs. Several beds have been endowed during the year. Mr. and Mrs. Frank Smart have endowed the "Frank and Marion bed" by a gift of £1,000 and Madame de Blumer has dedicated another to her son "Max de Blumer," especially for the longer treatment of young men suffering from diseases of the chest. The hospital has also been the recipient of several valuable gifts and legacies. The Convalescent Home at Eastbourne has been well occupied, and is financially satisfactory. It is to be regretted that the institution provides only for women and children. In the medical school a fresh development is the institution of a Hahnemann gold medal. This is to stimulate original research and discovery in homœopathic therapeutics. The medal, value £10, will be awarded every two years.

